# Electroporation Facilitates Introduction of Reporter Transgenes and Virions into Schistosome Eggs

**DOI:** 10.1371/journal.pntd.0000593

**Published:** 2010-02-02

**Authors:** Kristine J. Kines, Gabriel Rinaldi, Tunika I. Okatcha, Maria E. Morales, Victoria H. Mann, Jose F. Tort, Paul J. Brindley

**Affiliations:** 1 Department of Microbiology, Immunology & Tropical Medicine, George Washington University Medical Center, Washington, D.C., United States of America; 2 Department of Tropical Medicine, Tulane University Health Sciences Center, New Orleans, Louisiana, United States of America; 3 Departamento de Genética, Facultad de Medicina, Universidad de la República (UDELAR), Montevideo, Uruguay; 4 Tulane Cancer Center, Tulane University Health Sciences Center, New Orleans, Louisiana, United States of America; University of Queensland, Australia

## Abstract

**Background:**

The schistosome egg represents an attractive developmental stage at which to target transgenes because of the high ratio of germ to somatic cells, because the transgene might be propagated and amplified by infecting snails with the miracidia hatched from treated eggs, and because eggs can be readily obtained from experimentally infected rodents.

**Methods/Findings:**

We investigated the utility of square wave electroporation to deliver transgenes and other macromolecules including fluorescent (Cy3) short interference (si) RNA molecules, messenger RNAs, and virions into eggs of *Schistosoma mansoni*. First, eggs were incubated in Cy3-labeled siRNA with and without square wave electroporation. Cy3-signals were detected by fluorescence microscopy in eggs and miracidia hatched from treated eggs. Second, electroporation was employed to introduce mRNA encoding firefly luciferase into eggs. Luciferase activity was detected three hours later, whereas luciferase was not evident in eggs soaked in the mRNA. Third, schistosome eggs were exposed to Moloney murine leukemia virus virions (MLV) pseudotyped with vesicular stomatitis virus glycoprotein (VSVG). Proviral transgenes were detected by PCR in genomic DNA from miracidia hatched from virion-exposed eggs, indicating the presence of transgenes in larval schistosomes that had been either soaked or electroporated. However, quantitative PCR (qPCR) analysis determined that electroporation of virions resulted in 2–3 times as many copies of provirus in these schistosomes compared to soaking alone. In addition, relative qPCR indicated a copy number for the proviral luciferase transgene of ∼20 copies for 100 copies of a representative single copy endogenous gene (encoding cathepsin D).

**Conclusions:**

Square wave electroporation facilitates introduction of transgenes into the schistosome egg. Electroporation was more effective for the transduction of eggs with pseudotyped MLV than simply soaking the eggs in virions. These findings underscore the potential of targeting the schistosome egg for germ line transgenesis.

## Author Summary

The genome sequences of two of the three major species of schistosomes are now available. Molecular tools are needed to determine the importance of these new genes. With this in mind, we investigated introduction of reporter transgenes into schistosome eggs, with the longer-term aim of manipulation of schistosome genes and gene functions. The egg is a desirable developmental stage for genome manipulation, not least because it contains apparently accessible germ cells. Introduction of transgenes into the germ cells of schistosome eggs might result in transgenic schistosomes. However, the egg is surrounded by a thick shell which might block access to entry of transgenes. We cultured eggs in the presence of three types of reporter transgenes of increasing molecular size, and in addition we tried to produce transient holes in the eggs by electroporation to investigate whether the transgenes would more easily enter the eggs. Electroporation of eggs appeared to allow entry of two larger types of transgenes into cultured schistosome eggs, messenger RNA encoding firefly luciferase, and retroviral virions. We anticipate that this approach, electroporation of transgenes into schistosome eggs, will facilitate genetic manipulation of schistosomes for investigating the importance of schistosome genes and gene products as new intervention targets.

## Introduction

Advances in molecular genetics and immunology hold the promise to control the spread of schistosomiasis and to combat the morbidity and mortality associated with this neglected tropical disease [1]. Currently, control of schistosomiasis largely relies on chemotherapy with praziquantel, but its widespread use has led to concerns about development of drug resistance [2]. Schistosomes have comparatively large genomes, estimated at 398 megabase pairs (MB) for the haploid genome of *Schistosoma japonicum* [3] and 363 MB for *S. mansoni* [4]. Schistosome genes are arrayed on seven pairs of autosomes and one pair of sex chromosomes. *S. haematobium*, the other major schistosome species parasitizing humans probably has a genome of similar size, based on similarity of the karyotypes [5]. The schistosome genomes are the first to be published from among the Lophotrochozoa, an assemblage that includes about half of all animal phyla [6]. Analysis of the genomes revealed the presence of ∼13,000 protein-encoding genes, about 40% repetitive sequence content (retrotransposons, etc.), pervasive domain structure reduction, complex signal transduction and sensory pathways, proliferation of mini-exons, curious intron size distribution, large numbers of protease encoding genes, and other remarkable features [3],[4],[7].

Despite this abundance of sequence data, functional analysis of potential target genes will not be possible until reliable methods for reverse genetics in schistosomes become available. Transformation and gene manipulation in schistosomes have been reviewed recently (e.g., [8]–[10]. Schistosomes are large, multicellular eukaryotes, and though aceolomate, they possess complex organ systems including a blind gut with absorptive and secretory functions, well developed muscles, nervous tissues with complex sensory systems (like eyespots), and separate sexes with complex female and male reproductive tissues. The blood stage forms are covered by a syncytial tegument that is bordered at the parasite-host interface with a double lipid bilayer. Furthermore, the developmental stages differ dramatically in appearance and structure, cell numbers, ratio of germ to soma, and morphology. All these features pose challenges for genetic manipulation, and especially for germ line transgenesis. However, genetic manipulation and germ line transgenesis are worthwhile goals because they would facilitate a deep understanding of the molecular biology of schistosomes, roles of molecules in host-parasite interaction and, ultimately, to identify gene products that could be targeted/disrupted with drugs or vaccines.

Although the entire developmental cycle of the human schistosomes cannot be maintained *in vitro*, laboratory maintenance of the developmental cycles of all three human schistosomes can be accomplished using rodents as the mammalian host and the intermediate host snails [11],[12]. In addition, several developmental stages including mammalian and molluskan parasitic stages can be maintained *in vitro* (see [13]). Schistosome eggs can be obtained from livers of experimentally infected rodents, and miracidia obtained from these eggs are infectious for the intermediate host [14],[15]. In addition, the eggs can be maintained *in vitro* for at least one week and retain viability [16]–[19].

The schistosome egg represents an advantageous developmental stage of the schistosome at which to target transgenes because of its availability from experimentally infected rodents, high ratio of germ to somatic cells and because miracidia hatching from eggs can be employed to infect snails and propagate the developmental cycle. On the other hand, the developing miracidium is enclosed within an electron-dense, environmentally resistant egg shell [20]–[22]. Here we explored the introduction of transgenes and other macromolecules into eggs of *S. mansoni* by square wave electroporation. Square wave electroporation was more efficient than soaking alone for transduction of schistosome eggs by messenger RNA encoding luciferase and by pseudotyped retrovirus virions.

## Materials and Methods

### Developmental stages of *S. mansoni*

Mice infected with the NMRI (Puerto Rican) strain of *Schistosoma mansoni* were supplied by Dr. Fred Lewis, Biomedical Research Institute, Rockville, MD. Both adult worms and eggs were recovered from infected mice, as described [14], using a protocol approved by the Institutional Animal Case and Use Committee of The George Washington University. Eggs recovered from mouse livers were cultured for up to seven days at 37°C under 5% CO_2_ in air in Dulbecco's modified Eagle's medium (DMEM) supplemented with 10% fetal bovine serum, 100 U of penicillin and streptomycin [13],[19]. These eggs were washed three times in phosphate buffered saline, pH 7.4 (PBS) before exposure to Cy3-labeled siRNAs, mRNA, or virions. Virion-exposed eggs were transferred to sterile water under bright light to induce egg hatching and release of miracidia [13]. Miracidia were harvested from hatching eggs every 30 min for two hours. In some PCR based experiments, genomic DNAs, isolated from virion transduced sporocysts, and known to contain integrated proviral transgenes [23] were included as positive controls.

### Exposure of eggs to Cy3-labeled siRNA

*S. mansoni* eggs were either electroporated and soaked in non-coding Cy3-labeled siRNAs (Silencer Cy3-Labeled Negative Control siRNA, catalog no. AM4621, Ambion, Austin, TX) at 50 ng/µl with conditions recommended by Correnti et al. [24], or exposed similarly to Cy3-labeled siRNA without electroporation. Eggs were electroporated in 100 µl of schistosomule wash medium (RPMI 1640 with 200 U/ml penicillin G sulfate, 200 µg/ml streptomycin sulfate, 500 ng/ml amphotericin B, 10 mM HEPES) in 4 mm gap cuvettes with an ElectroSquarePorator ECM830 (BTX, San Diego, CA) using a single square wave pulse of 125 volts for 20 milliseconds. After electroporation, eggs were transferred into complete DMEM at 37°C. Three hours after exposure to Cy3-siRNA, with or without electroporation, eggs were washed in culture medium three times in order to remove the unincorporated Cy3-labeled siRNAs. Thereafter, they were observed under bright and fluorescent light (see below) using a Zeiss Axio Observer A.1 inverted microscope fitted with a digital camera (AxioCam ICc3, Zeiss). The eggs were cultured overnight and additional images collected 18 hours after electroporation and/or soaking. Manipulation of digital images was undertaken with the AxioVision release 4.6.3 software (Zeiss).

### Synthesis and delivery of luciferase mRNA

To synthesize firefly luciferase mRNAs (mFLuc), a template was prepared using PCR amplification of the luciferase gene in plasmid pGL3-Basic (Promega, Madison, WI) as described [25]. *In vitro* transcriptions of capped RNAs from DNA templates were accomplished using the mMessage mMachine T7 Ultra kit (Ambion, Austin, TX). Subsequently, ammonium acetate precipitated mFLuc was dissolved in nuclease-free water and quantified by spectrophotometry (ND-1000, NanoDrop Technologies, Wilmington, DE). *S. mansoni* eggs in culture for 48 h after isolation from mouse livers were subjected to electroporation in the presence of, and/or soaked in mFLuc at 100 and 130 ng/µl. Briefly, approximately 1,500–2,500 eggs were subjected to the square wave electroporation in 4 mm gap pathway cuvettes (BTX) in 120 µl of schistosomule wash medium [13],[25] using one 20 millisecond pulse of 125 volts. Thereafter, eggs were transferred to complete DMEM at 37°C, cultured for 3 h, or as indicated, washed three times in schistosomule wash medium and then stored as pellets at −80°C. Similar numbers of eggs were also soaked in complete DMEM with mFLuc at 130 ng/µl and maintained in culture for 3 h and washed three times before harvest.

### Luciferase activity assay

Luciferase activity in extracts of eggs was monitored using Promega's luciferase assay reagents [25],[26]. In brief, eggs were disrupted by sonication (5×5 sec bursts, output cycle 5, Misonix Sonicator 3000) (Misonix, Farmingdale, NY 11735) in 300 µl 1× CCLR lysis buffer (Promega). Aliquots of the egg sonicate (100 µl) were injected into 100 µl luciferin (Promega) at room temperature, mixed, and the relative light units (RLUs) determined ten seconds later at 560 nm in a Sirius tube luminometer (Berthold, Pforzheim, Germany). Duplicate samples were measured, with results presented as the average RLU readings per µg of soluble egg protein. The protein concentration in the soluble fraction of the egg lysate was determined using the bicinchoninic acid assay (BCA kit, Pierce, Rockford, IL). Recombinant firefly luciferase (Promega) was included as a positive control.

### Transduction of schistosome eggs with pseudotyped retrovirus

VSVG-pseudotyped virions were produced in GP2-293 cells transfected with plasmid constructs pLNHX-*Sm*ACT-Luc and pVSVG, as described [23]. pLNHX-*Sm*ACT-Luc includes the reporter gene encoding firefly luciferase (FLuc) under control of the actin gene promoter of *S. mansoni* [23],[27]. Viral supernatants were incubated with DNase I (New England Biolabs, Ipswich, MA) to remove any contaminating pLNHX plasmids using the method of Bruce et al [28]. After centrifugation, the pellet of concentrated virions was resuspended in Opti-MEM Reduced Serum Medium (Invitrogen). The viral titer was determined with a biological assay using target NIH-3T3 mouse fibroblast cells cultured in the presence of geneticin, as described [23].

Schistosome eggs were cultured in 35-mm tissue culture plates in ∼2 ml medium containing ∼200 µl of virions (VSVG-MLV) at 6×10^5^ colony forming units (cfu)/ml in the presence of 8 µg/ml polybrene (Sigma-Aldrich, St. Louis, MO). Other eggs exposed to the same VSVG-MLV inocula were also subjected to square wave electroporation using methods adapted from Pearce and coworkers [29]; the eggs were electroporated in 4 mm gap cuvettes in 400 µl of schistosomule wash medium and 200 µl of VSVG-MLV virions, using a single 125 V pulse of 20 milliseconds duration, as above. After electroporation, eggs were transferred into culture medium containing 8 µg/ml polybrene. Eighteen hours later, eggs were washed to remove virions and polybrene, and cultured for a further two days before hatching. Subsequently, genomic DNAs were isolated from the miracidia, and the presence or absence of the luciferase proviral transgene was investigated by direct PCR.

In two additional experiments, a quantitative PCR (qPCR) strategy was used to investigate whether electroporation could influence copy number of proviral transgenes. In the first experiment, eggs were cultured for two days before exposure, by electroporation or soaking, to ∼200 µl of a virion suspension (VSVG-MLV) at 2×10^4^ cfu/ml containing 8 µg/ml polybrene. Eggs were cultured for three days after virion exposure, after which they were transferred water to induce release of miracidia. For the second qPCR experiment, eggs were cultured for three days before exposure by electroporation or soaking to the virion suspension described above, cultured for two more days after virion exposures, and then eggs transferred to water to induce release of miracidia. In both experiments, at one day after exposure to virions, eggs were washed in culture medium to remove virions and polybrene. gDNAs were isolated from the miracidia, and employed as templates for qPCR analysis of transgene copy numbers.

### Detection of provirus in transduced schistosomes

Total genomic DNA (gDNA) was isolated from transduced and control untreated developmental stages of schistosomes, including mixed sex adult worms, using the AquaPure system (Bio-Rad, Hercules, CA). In order to investigate the presence of double stranded, proviral transgenes, we employed gDNAs isolated from miracidia hatched from transduced eggs as templates for direct PCR using the firefly luciferase primers 5′-GGAGAGCAACTGCATAAGG and 5′-AATCTCACGCAGGCAGTTCT (see below). As a positive control for the PCR, we amplified the *S. mansoni* cytochrome oxidase I (*cox I*) gene (GenBank AF101196, using *cox 1*-specific primers 5′-TGAGTGTCATTTTAGGGTGGTG and 5′-ACAAACCAATGAAAATATCCAAGA) which we have shown previously to be amplified from these kinds of gDNA preparations [27]. In addition, as negative controls, we included templates of gDNA from non-virion exposed adult schistosomes and/or included reactions where water was substituted for gDNA. PCRs were carried out using Master Mix (Promega) reagents, and 35 thermal cycles of 94°C, 1 min, 50°C, 1 min, and 72°C, 2 min. Amplification products were separated by electrophoresis through 1% agarose, stained with ethidium bromide, visualized under UV illumination and digital images captured (Gene-Doc, Bio-Rad). After electrophoresis to determine their sizes, PCR products were Southern blotted onto Zeta-Probe (Bio-Rad) nylon. A ∼5.3 kb *Kpn* I fragment pLNHX-*Sm*ACT-Luc (including the luciferase coding sequence) [27] was isolated, labeled with ^32^P.dCTP by random oligomer priming (RadPrime, Invitrogen) [27] and used as a probe. Southern blots were hybridized at 65°C to the labeled probe for 18 h, washed at high stringency [30], and hybridization signals detected by autoradiography on Biomax film (Kodak).

### Real-time quantitative PCR and estimation of transgene copy numbers

Primers were designed with the assistance of Beacon Designer (Premier Biosoft International, Palo Alto, CA) to obtain primer and TaqMan probe sequences targeting the firefly luciferase (FLuc) (from pGL3-Basic, Promega, Madison, WI) and *S. mansoni* cathepsin D (*Sm*CathD) (GenBank U60995) genes, as follows: for FLuc, forward primer: 5′-TGC TCC AAC ACC CCA ACA TC-3′; reverse primer: 5′- ACT TGA CTG GCG ACG TAA TCC-3′; probe: 5′-/56-FAM/ACG CAG GTG TCG CAG GTC TTC C/3IABlk_FQ/-3′; for *Sm*CathD, forward primer : 5′-TGG GCT CAC TGA GTG TAA AGG-3′; reverse primer: 5′-CAT ACC AAG GAT ACC ATC GAA CTT C-3′; probe: 5′-/56-FAM/ACC CTG GTT GTT GTG TCG CTT CCC/3IABlk_FQ/-3′. Quantitative PCRs were performed in triplicate, using 96-well plates (Bio-Rad), with al denaturation step at 95°C from 3 minutes followed by 40 cycles of 30 sec at 95°C and 30 sec at 55°C, using a thermal cycler (iCycler, Bio-Rad) and a Bio-Rad iQ5 detector to scan the plates in real time. Reactions were carried out in 20 µl volumes with primer-probe sets (FLuc, *Sm*CathD) and Perfecta qPCR FastMix, UNG (Quanta Bioscience, Gaithersburg, MD).

Absolute quantification was undertaken using 250 ng of gDNA samples or copy number standards, i.e. 10-fold serial dilutions of pGL3, from 1.93×10^3^ copies to 1.93×10^10^ copies. The exact copy number of each diluted plasmid was calculated through the relationship between the molecular mass of pGL3 and the Avogadro constant, *N*_A_. Absolute copy number of the luciferase transgene per ng of schistosome gDNA was estimated by interpolation of the sample PCR signals from a standard curve (see [31]).

Relative quantification was performed in order to estimate the transgene copy number in comparison with an endogenous schistosome gene of known copy number [31],[32] in particular the single copy number gene, *Sm*CathD. *Sm*CathD encodes the cathepsin D aspartic protease of *S. mansoni* that participates in hemoglobin proteolysis [33]. The PCR efficiencies for the FLuc transgene and the *Sm*CathD gene were estimated by titration analysis [31] to be 99.0% and 97.3%, respectively (not shown). Five 10-fold decreasing serial dilutions starting from 200 ng of gDNA of each sample were used as templates to target the *Sm*CathD and FLuc genes, in different reactions. Estimation of the relative copy number of FLuc was derived from ΔCt values for *Sm*CathD. To calculate ΔCt values, the average of triplicate Ct values generated with the luciferase primers-probe set was subtracted from the average *Sm*CathD Ct values. The copy number ratio between *Sm*CathD and FLuc in each sample was obtained with the equation, 2^ΔCt^ [31].

### Statistical analysis

Statistical differences among and between groups were investigated using analysis of variance (ANOVA) and Student's *t*-test. *P*-values of ≤0.05 were considered to be significant.

## Results

### Cy3-labeled siRNA penetrates into *S. mansoni* eggs

**Figure 1:**
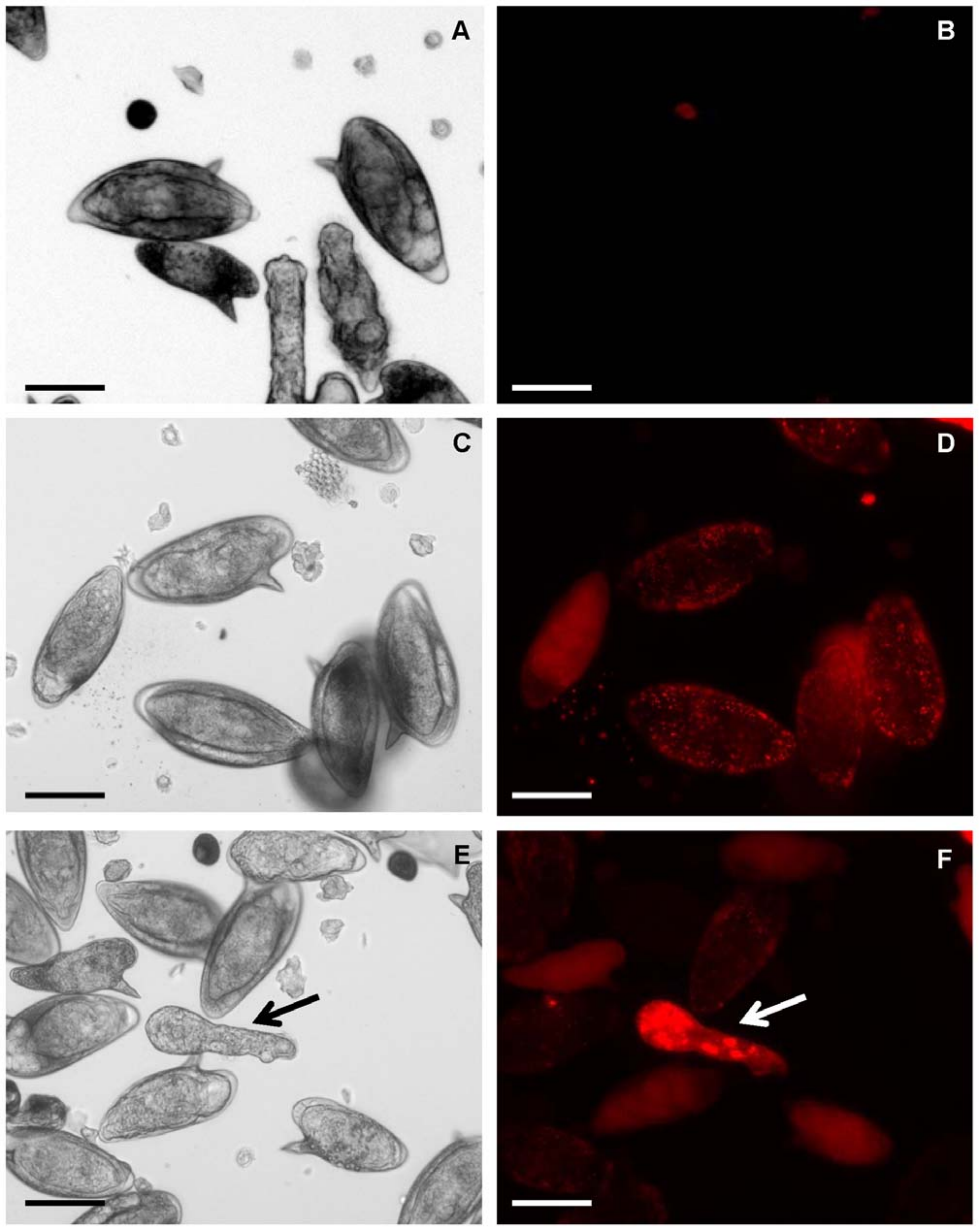
Fluorescent labeled short interfering RNA enters cultured eggs of Schistosoma mansoni. Representative images of schistosome eggs and miracidia 24 hours after soaking in Cy3-siRNA; bright field, upper panels, fluorescence, lower panels. Eggs were soaked in medium containing 50 ng/µl of Cy3-siRNA. No Cy3-siRNA treatment control (A, B), Cy3-siRNA treated, fluorescent eggs (C, D) and fluorescent eggs and a miracidium/sporocyst (arrow in E, F). Scale bar, 50µm.

**Figure 2:**
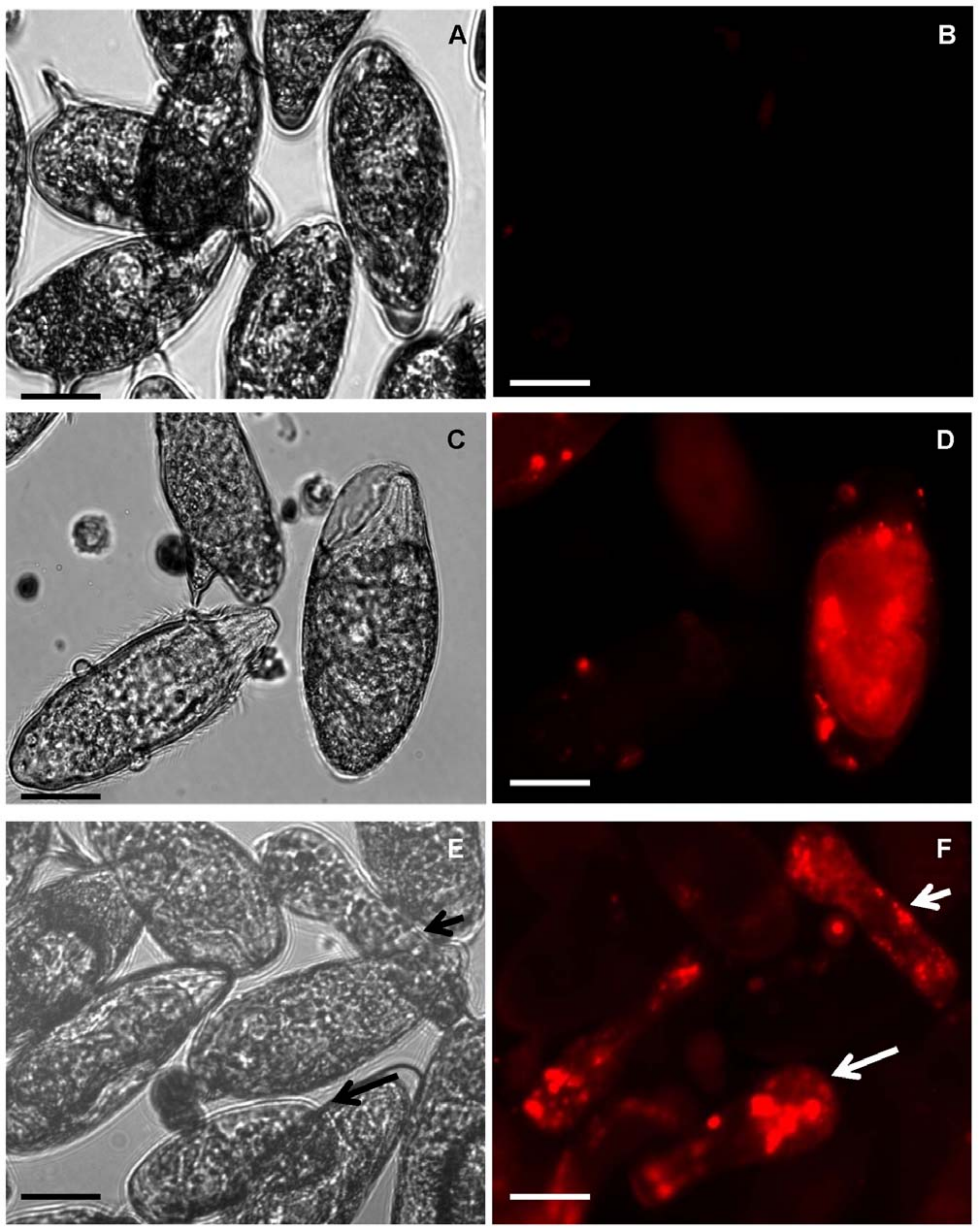
Fluorescent labeled short interfering RNA enters cultured eggs of *Schistosoma mansoni* after square wave electroporation. Representative images of schistosome eggs and miracidia 24 hours after electroporation with Cy3-siRNA; bright field, upper panels; fluorescence field, lower panels. Eggs were electroporated in medium containing 50 ng/µl of Cy3-siRNA. No Cy3-siRNA treatment control (A, B), Cy3-siRNA treated, fluorescent eggs (C, D) and fluorescent eggs and miracidium/sporocyst (arrows in E, F). Scale bar, 20µm.

To investigate whether macromolecules could penetrate into *S. mansoni* eggs, cultures of schistosome eggs were incubated in a Cy3-siRNA (∼13.8 kDa) with or without concomitant square wave electroporation. Three hours after exposure to Cy3-siRNA, eggs were examined by fluorescence microscopy which revealed diffuse but weak fluorescence within the eggs (not shown). By contrast, by 18 hours after soaking or electroporation with Cy3-siRNA, eggs displayed strong fluorescence including foci of intense fluorescence (Figures 1, 2). Many eggs had hatched, releasing miracidia; the miracidia displayed multiple intense areas of fluorescence, indicating uptake of Cy3-siRNA by the eggs (Figures 1, 2, panels e, f in both). Either soaked or electroporated eggs with Cy3-siRNA and the corresponding hatched miracidia/sporocysts showed intense fluorescence. In contrast, control eggs treated similarly but without Cy3-siRNA, i.e. mock treatment controls, showed no specific fluorescence (Figure S1). Soaked eggs displayed spots of strong fluorescence throughout the eggs (Figure 1, panel d; Figure S2, panel b). Miracidia/sporocysts hatched from the Cy3-siRNA exposed eggs often exhibited intense signals, with large, bright fluorescent foci (Figure 1, panel f). Compared to eggs soaked with Cy3-siRNA, electroporated eggs displayed a more diffuse Cy-3 fluorescence (Figure 2, panel d and Figure S3, panel b), contained within the egg shell.

The miracidia/sporocysts hatched from electroporated eggs displayed intense foci of fluorescence (Figure 2, panel f; Figure S3, panel d), similar to the Cy3-siRNA soaked groups. The signal was distributed throughout the entire larval body, but often with foci of strong fluorescence at the posterior extremity. Lack of fluorescence signal in the ciliated plates shed from the miracidia indicated that the Cy3-siRNA was incorporated into the larvae and was not retained in the surface (Figure S3, panel d). These data indicated that the electroporated Cy3-siRNA entered the eggs, perhaps traversing through the cribriform pores of the eggshell [22], and entered the miracidium within the eggshell. Neither soaked nor electroporated control eggs displayed fluorescence (Figure 1, panel b; Figure 2, panel b).

### Reporter gene luciferase mRNA electroporated into schistosome eggs

**Figure 3:**
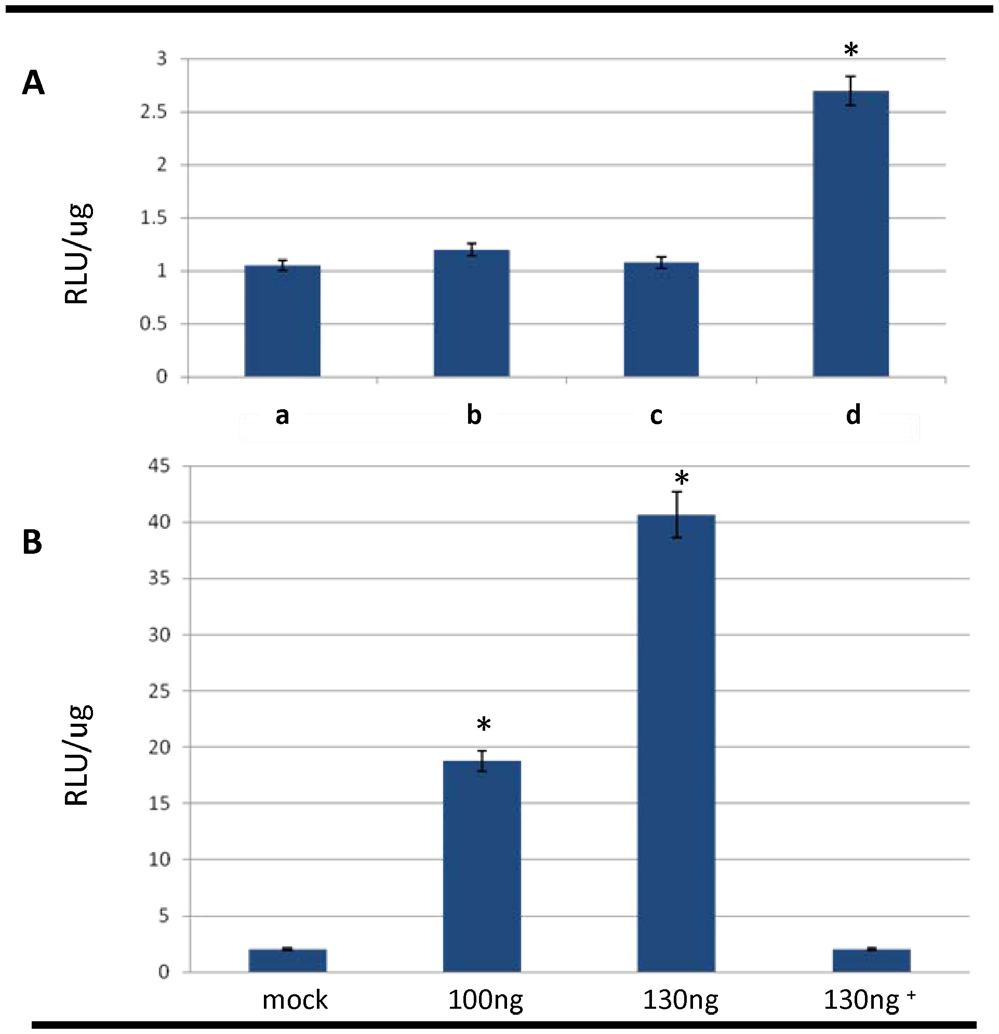
Luciferase activity in *Schistosoma mansoni* eggs. Detection of luciferase activity in extracts of eggs treated with capped mRNA encoding firefly luciferase (mFLuc). Panel A: luciferase activity three hours after soaking or electroporation with 130 ng/µl of mFLuc; a, negative controls soaked without mFLuc; b, eggs soaked with mFLuc; c, negative control eggs electroporated without mRNA; d, eggs electroporated with mFLuc. RLUs/µg, relative light units per microgram of egg protein. Panel B: luciferase activity three hours after electroporation of eggs in control (mock) and experimental groups with 100ng/µl and 130ng/µl of mFLuc, and at 30 hours after electroporation with 130 ng/µl (indicated with cross symbol). Asterisks denote statistically significant differences (*P*≤0.05) among groups.

To investigate whether transgene mRNAs could penetrate schistosome eggs, we soaked and/or electroporated cultured eggs in firefly luciferase mRNA (mFLuc) (∼512 kDa). More specifically, after two days in culture, 1,500–2,500 eggs were soaked or electroporated with 130 ng/µl of mFLuc; the eggs were collected three hours later. Luciferase activity was measured, with relative luminescence units (RLUs/µg) normalized per µg of soluble protein extracted from the eggs. Significant luciferase activity was detected in the mFLuc electroporated group compared with the others (*P*<0.05) (Figure 3A). By contrast, no significant differences were apparent among the other treatment groups. Because significant luciferase activity was observed only in eggs electroporated with mFLuc, we investigated the influence of increasing concentrations of mFLuc, 0 ng/µl, 100 ng/µl, and 130 ng/µl, and in eggs harvested three hours after electroporation. Significant luciferase activity was observed in homogenates of the transformed eggs with all three concentrations of mFLuc whereas untreated worms show negligible activity (*P*<0.05) (Figure 3B). Since mRNAs usually exhibit short half lives *in vivo*, we also examined luciferase activity at 30 hours after electroporation of the eggs with 130 ng/µl, as an indirect measure of mFLuc stability. Little or no luciferase was detected at 30 hours after electroporation (Figure 3B). Collectively, these findings indicated that square wave electroporation efficiently delivered exogenous nucleic acids into the eggs of *S. mansoni*.

### Schistosome eggs transduced by pseudotyped retroviral virions

**Figure 4:**
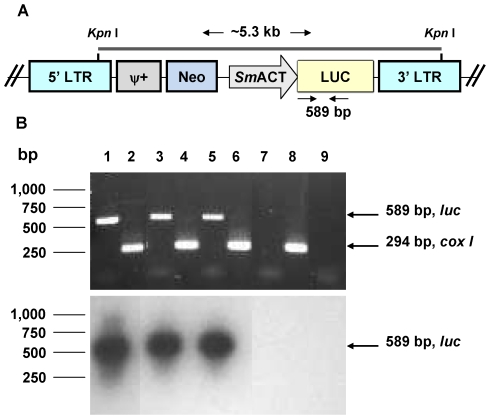
Detection by end-point PCR of retroviral transgenes in miracidia hatched from virion-transduced eggs. Panel A: Schematic representation of retroviral construct pLNHX-*Sm*ACT-Luc, showing position of *Kpn* I fragment employed as the hybridization probe. The retrovirus cassette included the firefly luciferase reporter gene (LUC) driven by the *S. mansoni* actin 1.1 gene promoter (*Sm*ACT), flanked by the 5′ and 3′ long terminal inverted repeats of the murine leukemia virus (5′LTR and 3′LTR). The cassette also included the gene endowing neomycin resistance (Neo) and the psi motif (ψ^+^), involved in packaging the viral DNA). Panel B: Top panel: ethidium-stained PCR products resolved in agarose gel. Genomic DNAs (gDNA) from miracidia hatched from eggs transduced with pLNHX-*Sm*ACT-Luc virions were employed as templates for PCR using primers specific for *luc* transgene (lanes 1, 3, 5, 7) and *cox I*, a positive control endogenous schistosome gene, (lanes 2, 4, 6, 8). A reaction without template gDNA with primer pairs specific for the *luc* gene served as the negative control (lane 9). A gDNA sample (from transduced sporocysts) known to be positive for integrated transgenes was included as the positive PCR control for *luc* and *cox I* (lanes 1, 2). The miracidia analyzed in lanes 3 (*luc*) and 4 (*cox I*) were hatched from eggs electroporated in virus, and the miracidia analyzed in lanes 5 (*luc*) and 6 (*cox I*) were hatched from eggs soaked in virus. gDNA from non-transduced, control adults (negative control) were used as template for lanes 7 (*luc*) and 8 (*cox I*). Molecular size standards in base pairs (kb) are shown at the left, while the sizes of signals for *luc* (589 bp) and *cox 1* (294 bp) are indicated at the right. Bottom panel: autoradiograph of Southern hybridization signals from the PCR products (visualized in top panel) to a radiolabeled probe, a ∼5.3 kb *Kpn* I fragment of pLNHX-*Sm*ACT-Luc spanning the genes encoding neomycin resistance (*neo*) and firefly luciferase (*luc*) (panel A).

Schistosome eggs were electroporated and/or soaked in the presence of VSVG pseudotyped pLNHX-*Sm*ACT-Luc virions. One to three days later, eggs were incubated in sterile water to induce hatching of miracidia from the virion exposed eggs. We investigated whether these miracidia from eggs exposed to virions had been transduced by the retrovirus. Direct PCR analysis of gDNA isolated from miracidia from transduced eggs was employed to detect the presence of proviral retrovirus (schematic of predicted transgene provirus presented as Figure 4 A). As the positive control for the experiment, a 589 bp fragment of the reporter transgene encoding luciferase was amplified from gDNA from sporocysts known (from our previous studies [23],[34]) to contain integrated proviral transgenes *luc* (Figure 4B, lane 1). Likewise the *cox I* signal of 294 bp was amplified from the sporocyst gDNA (lane 2), indicating the integrity of the PCR. Furthermore, the *luc* transgene was also detected in miracidia from eggs that were either electroporated (lane 3) or soaked (lane 5) in pLNHX-*Sm*ACT-Luc virions. The control *cox 1* gene fragment of 294 bp also was amplified from these gDNAs, verifying the integrity of the templates (Figure 4B, lanes 4, 6). No FLuc gene specific amplification was seen using template gDNA from control worms not exposed to virions (lane 7) whereas the target 294 bp region of the *cox 1* gene was amplified (lane 8) from this control gDNA. The identity of PCR products as specific for the FLuc transgene was confirmed by Southern hybridization analysis to a labeled pLNHX-*Sm*ACT-Luc/*Kpn* I gene probe (Figure 4A). (These PCR findings demonstrated the presence of proviral transgenes within the treated larvae. We anticipate that many of the proviral transgenes had integrated into schistosome chromosomes, based on our earlier findings [27]. Whether or not the provirus had actually integrated or remained as non-integrated provirus does not negate the finding that electroporation was more efficient than soaking for transduction of schistosome eggs (below). However, Southern hybridization analysis of gDNA of a representative group of virion exposed eggs/miracidia indicated that proviral transgenes had integrated into the schistosome chromosomes (not shown).

It was noteworthy that miracidia hatched from the eggs soaked in pseudotyped virions did not appear to have lost vitality because of virion exposure. By contrast, many miracidia that hatched from electroporated eggs were less active; their movement was sluggish compared to miracidia from soaked eggs. Also, many eggs failed to hatch after electroporation (data not shown).

### Retroviral transduction of schistosome eggs facilitated by electroporation

**Figure 5:**
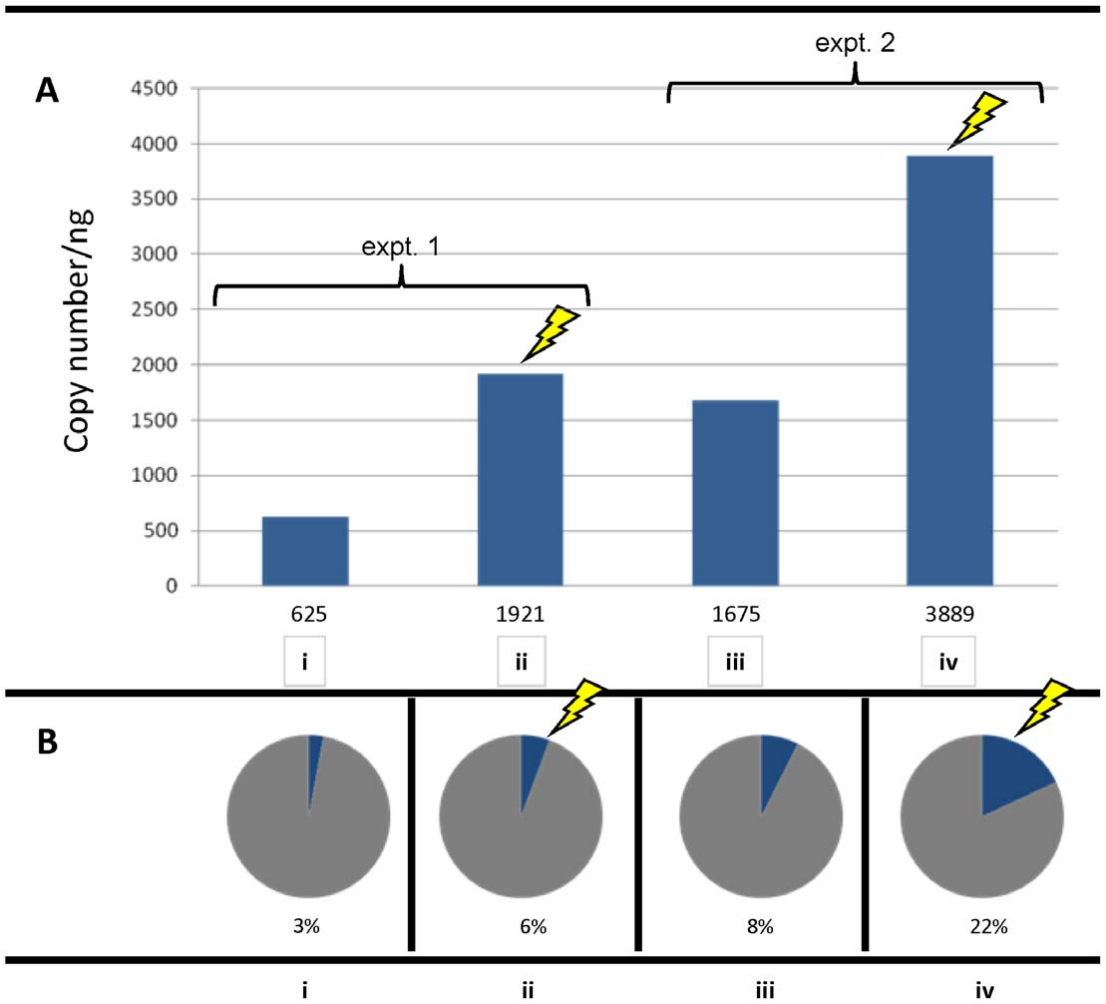
Copy numbers of luciferase transgenes ascertained by quantitative PCR. Panel A: Absolute copy number of the firefly luciferase (FLuc) transgene per ng of genomic DNAs from miracidia hatched from virion-exposed eggs - experiment (expt.) number 1 (i, soaking; ii, electroporation), experiment number 2 (iii, soaking; iv, electroporation). The absolute copy numbers are indicated below the bars. Panel B: Relative FLuc transgene copy number in comparison to the control *Sm*CathD (cathepsin D) single copy gene; the percentages represent the copy number of FLuc for every 100 copies of the cathepsin D gene. Transduced eggs from experiment no. 1 (i, soaking; ii, electroporation) and from experiment no. 2 (iii, soaking; iv, electroporation). The lightning flashes indicate treatment with electroporation.

Quantitative PCR (qPCR) was employed to determine the copy number of the proviral luciferase transgene in gDNAs from miracidia hatched from virion-exposed eggs. Methods yielding both absolute and relative quantification were used. Figure 5 summarizes the results from two related experiments. Eggs that had been in culture for 48 h (experiment no. 1) and eggs in culture for 72 h (experiment no. 2) were electroporated or soaked with pseudotyped MLV. Three days (no. 1) and two days (no. 2) later, gDNA was isolated from miracidia hatched from the eggs and assayed for the presence of the luciferase transgene by qPCR. In the first, about three times as many copies, and in the second experiment, more than twice as many copies were seen in the electroporated group compared to the soaked group (Figure 5A).

In order to estimate the ratio between the copy number of the transgene and that of a single-copy gene, we performed a relative quantification by qPCR. For every sample we performed real time PCR targeting luciferase and *Sm*CathD, a representative single copy gene. We saw a range of ratios among the four groups of gDNAs in both experiments 1 and 2, ranging from 0.03 copies of the FLuc transgenes for each copy of *Sm*CathD (no. 1, soaked) to 0.22 copies (no. 2, electroporated) (Figure 5B). More specifically, in each of the two experiments, the copy number of FLuc was 2 to 3 times higher in the electroporated group than in the soaked group. Together, these data indicate that square wave electroporation is more effective than soaking alone for delivering VSVG-MLV virions into eggs of *S. mansoni*.

## Discussion

The schistosome egg represents an attractive developmental stage at which to target transgenes because it is readily obtained from experimentally-infected rodents or naturally infected people, is easily maintained in vitro, has a high ratio of germ to somatic cells and contains miracidia that can be employed to infect snails to propagate the life cycle. Furthermore, from the clinical perspective, the egg represents the major source of pathogenesis in human schistosomiasis. Here we observed that exogenous macromolecules penetrate into cultured eggs, and we speculate that small macromolecules such as Cy3-Silencer siRNA (13.8 kDa) readily enter eggs through the pores that anastomose throughout the eggshell and which provide access from sub-shell envelope and the developing miracidium to the exterior [21],[22]. Interestingly, after exposure to fluorescent siRNA, strong foci of fluorescence were distributed at the posterior of the larva, where the germinal cells are located [17]. This suggests that germinal cells can be reached by reporter transgenes introduced into schistosome eggs.

Luciferase activity was detected in extracts of eggs three hours after electroporation of capped mRNA, but not after soaking alone. This outcome may reflect the labile nature of the luciferase mRNA, with quick entry of the mRNA into eggs precipitated by electroporation allowing translation before mRNA degradation. At 512 kDa/1652 nt, mRNA encoding firefly luciferase is a far larger macromolecule than Cy3-siRNA. We also electroporated eggs in the presence of VSVG-MLV virions, a massive particle of >10^8^ kDa [35]. Proviral MLV transgenes were detected in the miracidia and eggs using direct end-point PCR and qPCR.

The MLV virion is ∼100 nm in diameter [36], whereas the diameter of the cribriform pores on the surface of the schistosome eggs is ∼34 nm [22]. Thus it was remarkable that the virions apparently entered the eggs. In addition, beneath the eggshell there is an outer envelope, Reynold's layer, comprised of a fibrous matrix and a cellular inner envelope (von Lichtenberg's envelope) surrounding the developing miracidium [17],[21],[22],[37]. Serpiginous branching channels from the eggshell pores traverse the eggshell allowing molecules to cross the eggshell barrier, as shown by the soaking of dsRNA [16],[19]. Perhaps the electroporation causes an expansion of the diameter of the natural cribriform pores, or even establishes transient pores in the egg shell itself [38],[39], through which the virions and mRNA can be propelled into the eggs. In single cell systems, reversible membrane breakdown accompanies electroporation, providing the pulse time is brief. Under these conditions, short-lived perturbations (electropores) can form in membranes, allowing transient access to the cytosol. The electropores reseal quickly at 37°C, but permit ingress of macromolecules and particles including hormones, proteins, RNA, DNA and organelles without deterioration of cellular functions [38],[39]. Accordingly, electroporation may have produced electropores in the eggshell, the subshell envelope and/or cells of the developing miracidia through which the transgenes and/or virions entered cells of the schistosome larva. Even if electroporation ruptured or otherwise damaged the eggs, sufficient integrity may have been retained in many of them to allow the transformed miracidium to successfully hatch.

Quantitative real time PCR (qPCR) has been validated as a tool to ascertain transgene copy number and is as sensitive as Southern and dot blot hybridization [32],[40],[41]. We employed qPCR to estimate the copy number of the luciferase transgene and thereby evaluate the transduction efficiency of VSVG-MLV virions introduced into cultures of schistosome eggs by electroporation compared to soaking. The absolute quantification revealed the presence of 2–3 times more copies of the transgene in the electroporated compared to soaked eggs, indicating that electroporation was more efficient than soaking for transducing schistosome eggs. The outcome of the relative qPCR analysis was consistent with findings for absolute copy number of the transgene. Thus, since *S. mansoni* is diploid, somatic cells have two copies of each autosomal gene. Given that *Sm*CathD gene is a single copy gene [33], and that electroporation lead to the presence of ∼20 copies of the transgene for every 100 copies of *Sm*CathD, i.e. a transgene copy number of 0.2, we speculate that 20 copies of the luciferase transgene were distributed in every 50 cells. However, we do not yet know how many copies of the transgene were present in any specific cell, genome or indeed egg.

Ascertainment of relative copy number of the transgene in comparison to the copy number of an endogenous gene would be informative and diagnostic in approaches for germ line transgenesis. A relative copy number of ≥1, comparing the transgene with an endogenous single copy gene, is expected for transgenic organisms where all the cells will include at least one copy of the transgene. By contrast, the copy number of ∼0.2 we observed here reflects the situation that the transgene was not present in every cell of the transduced population of schistosome eggs. Indeed, we consider that most of the luciferase genes would have been located in cells at the periphery of the developing miracidium because these cells would be more likely to be transduced by the electroporated virions than cells deeper within the larva. (VSVG-MLV virions are replication deficient – after transduction of the cell, no virus is produced and so neighboring and/or deeper tissues remain uninfected.) In addition, since there is a high ratio of germ cells to somatic cells in the egg, and given that the location of the germ cells in the mature eggs has been established [17], it would be advantageous to introduce as many copies as possible of the transgene into this developmental stage in order to increase the likelihood of germ line integration.

These findings represent the first report of the utility of square wave electroporation for the introduction of exogenous macromolecules and virions into the schistosome egg. The egg/miracidia stages are attractive targets for transgenesis because they are rich in germ line cells. The transgenes may enter the eggs through the cribriform pores known to form networks from the exterior of the eggshell, and/or through electropores in as yet undetermined sites in the eggshell or surfaces of cells of the developing miracidium. In any event, these approaches confirm the egg stage as a tractable target for germ line transgenesis. They also are of potential use for investigating novel therapeutic interventions since eggs trapped in liver, and other organs, are the direct agents of pathogenesis in schistosomiasis.

## Supporting Information

Figure S1 Representative low magnification images (5×) of *Schistosoma mansoni* eggs and miracidia in culture 24 hours after exposure to Cy3-siRNA. (A) Eggs in culture soaked in Cy3-siRNA, 50 ng/µl. Mock control without Cy3-siRNA (a, bright field; b, fluorescence field), and Cy3-siRNA treated eggs and miracidia (c, bright field; d, fluorescence field). (B) Eggs electroporated in the presence of 50 ng/µl of Cy3-siRNA. Mock control without Cy3-siRNA (a, bright field; b, fluorescence field), Cy3-siRNA treated eggs and miracidia (c, bright field; d, fluorescence field). Scale bar, 100 µm.
(0.60 MB PDF)

Figure S2 Representative high magnification images (40×) of *Schistosoma mansoni* eggs, miracidia and sporocysts in culture 24 hours after soaking with Cy3-siRNA are shown. (A, B) (Bright and dark fields, respectively) Representative images of two eggs, one of them exhibiting fluorescent spots within the larvae. (C, D) (Bright and dark fields, respectively) Representative images of an egg, miracidium and sporocyst. Arrowhead, ciliated plate shed from a miracidium. Spo, sporocyst, Mir, miracidium. Scale bar, 20 µm.
(0.51 MB PDF)

Figure S3 Representative high magnification images (40×) of *Schistosoma mansoni* eggs, miracidia and sporocysts in culture 24 hours after electroporation with Cy3-siRNA are shown. (A, B) (Bright and dark field, respectively) Representative images of eggs, one of them with fluorescent spots within the larvae (white arrow). (C, D) (Bright and dark field, respectively) Images of an egg, miracidium and sporocyst. Arrowhead, ciliated plate shed from a miracidium. Spo, sporocyst, Mir, miracidium. Scale bar, 20 µm.
(0.47 MB PDF)
