# Supplementary figures and images for: Electroporation Facilitates Introduction of Reporter Transgenes and Virions into Schistosome Eggs
Source: PLoS Negl Trop Dis. 2010 Feb 2;4(2):e593. doi: 10.1371/journal.pntd.0000593 (PMC2814865; doi:10.1371/journal.pntd.0000593)

Figure S1

**A**

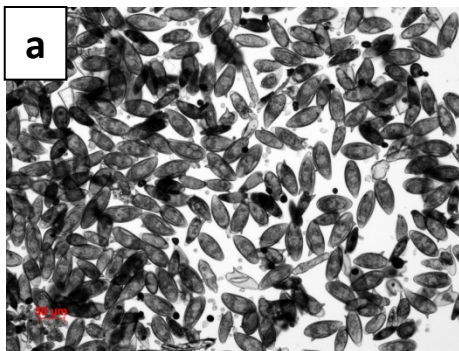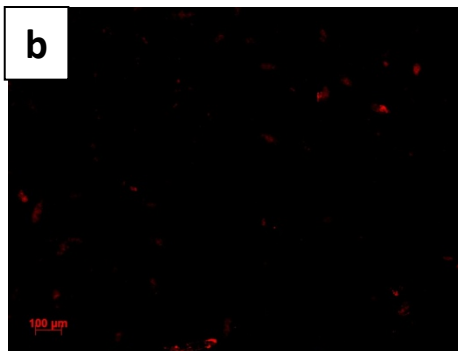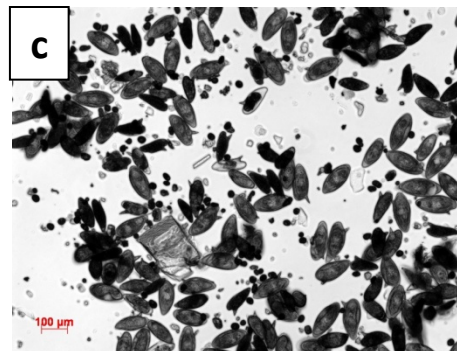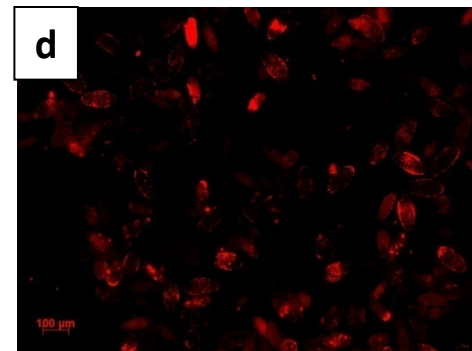

**B**

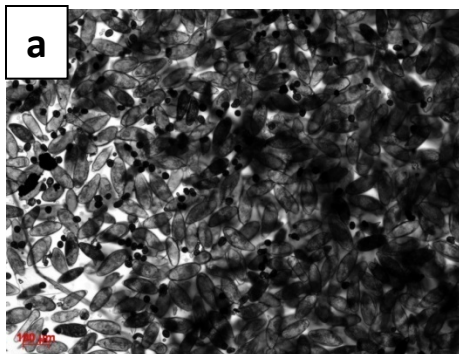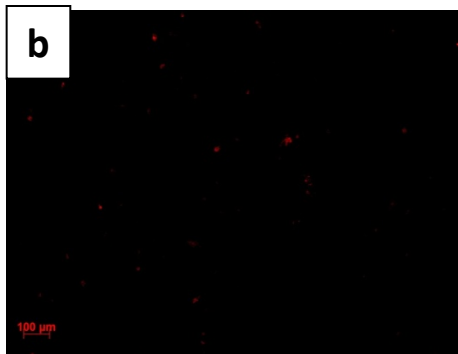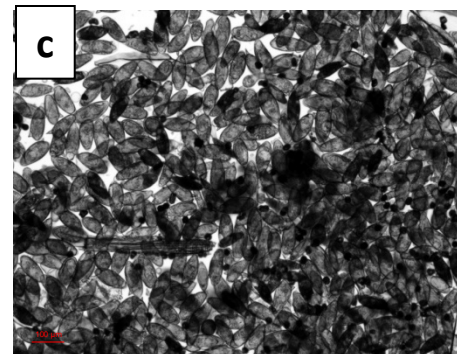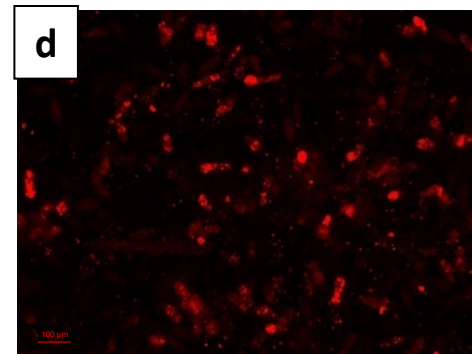

Supplement: Figure S1 — Representative low magnification images (5×) of Schistosoma mansoni eggs and miracidia in culture 24 hours after exposure to Cy3-siRNA. (A) Eggs in culture soaked in Cy3-siRNA, 50 ng/µl. Mock control without Cy3-siRNA (a, bright field; b, fluorescence field), and Cy3-siRNA treated eggs and miracidia (c, bright field; d, fluorescence field). (B) Eggs electroporated in the presence of 50 ng/µl of Cy3-siRNA. Mock control without Cy3-siRNA (a, bright field; b, fluorescence field), Cy3-siRNA treated eggs and miracidia (c, bright field; d, fluorescence field). Scale bar, 100 µm. (0.60 MB PDF) [file pntd.0000593.s001.pdf]

Figure S2

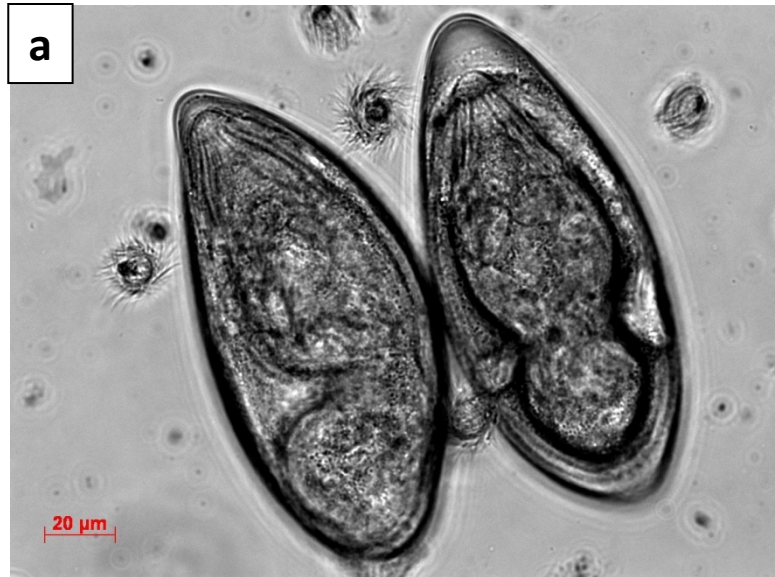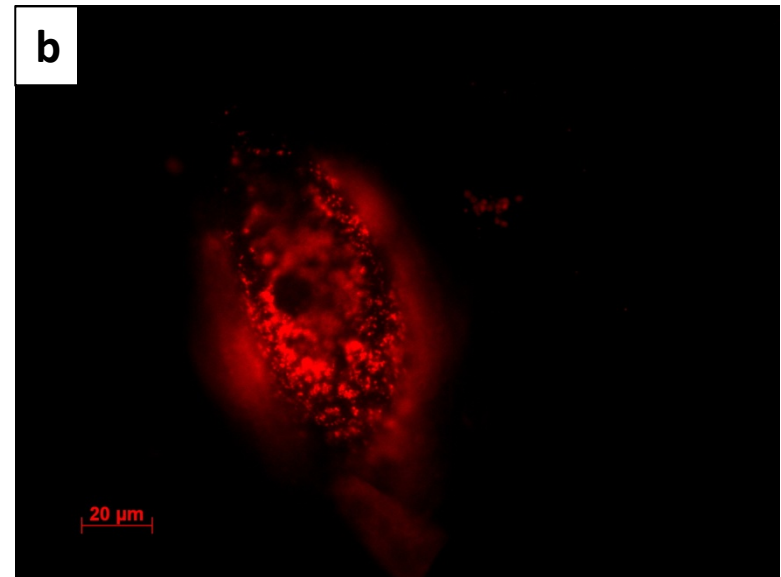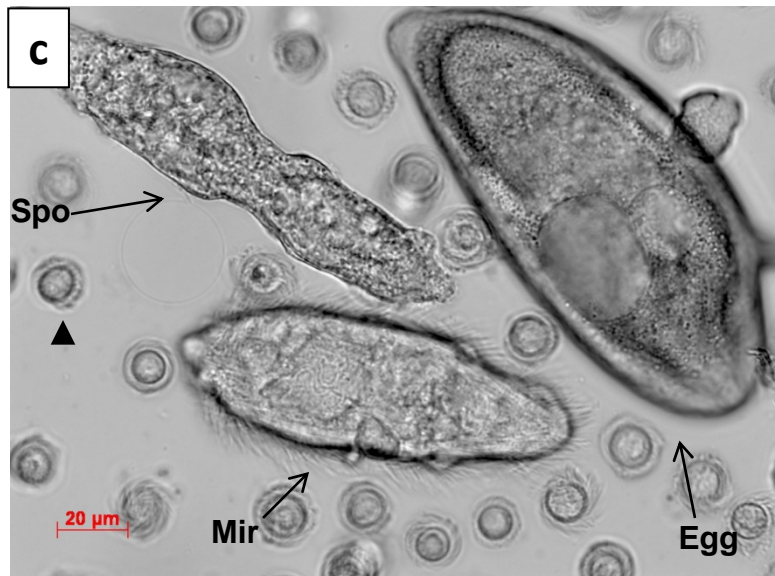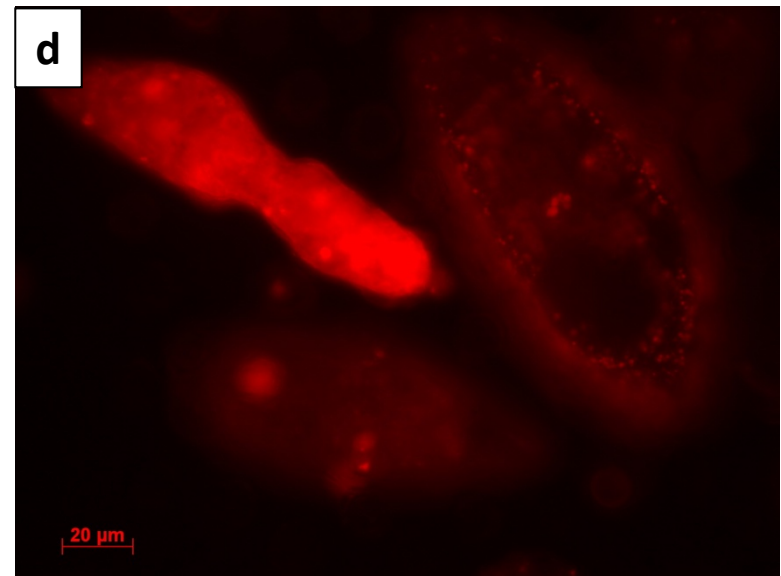

Supplement: Figure S2 — Representative high magnification images (40×) of Schistosoma mansoni eggs, miracidia and sporocysts in culture 24 hours after soaking with Cy3-siRNA are shown. (A, B) (Bright and dark fields, respectively) Representative images of two eggs, one of them exhibiting fluorescent spots within the larvae. (C, D) (Bright and dark fields, respectively) Representative images of an egg, miracidium and sporocyst. Arrowhead, ciliated plate shed from a miracidium. Spo, sporocyst, Mir, miracidium. Scale bar, 20 µm. (0.51 MB PDF) [file pntd.0000593.s002.pdf]

Figure S3

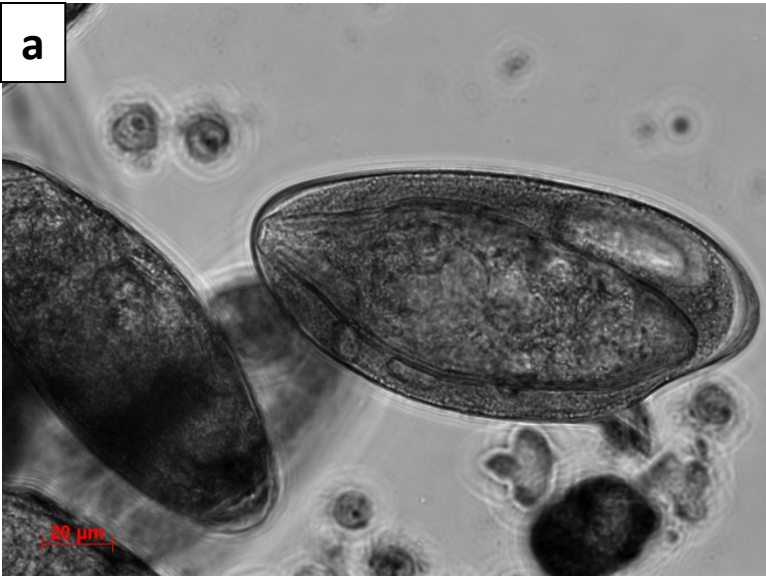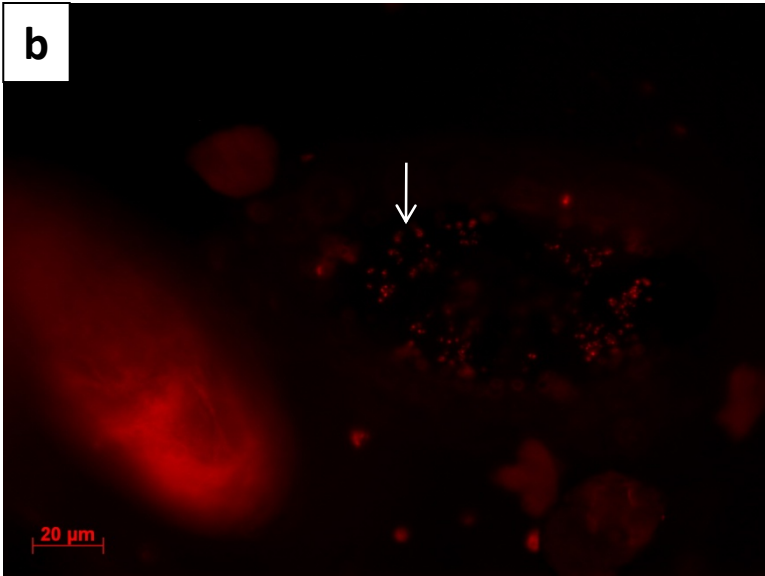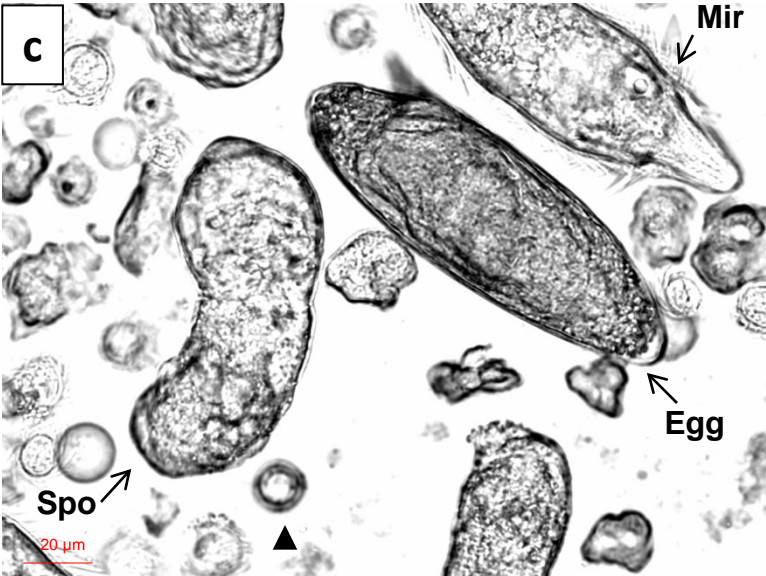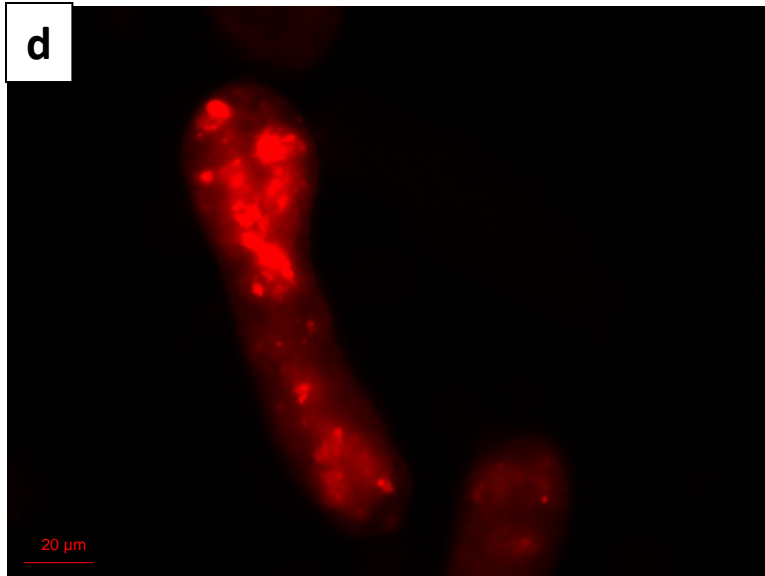

Supplement: Figure S3 — Representative high magnification images (40×) of Schistosoma mansoni eggs, miracidia and sporocysts in culture 24 hours after electroporation with Cy3-siRNA are shown. (A, B) (Bright and dark field, respectively) Representative images of eggs, one of them with fluorescent spots within the larvae (white arrow). (C, D) (Bright and dark field, respectively) Images of an egg, miracidium and sporocyst. Arrowhead, ciliated plate shed from a miracidium. Spo, sporocyst, Mir, miracidium. Scale bar, 20 µm. (0.47 MB PDF) [file pntd.0000593.s003.pdf]
